# Graph theory-based machine learning for automated fibromyalgia diagnosis using fMRI during an emotional task

**DOI:** 10.1371/journal.pone.0357964

**Published:** 2026-10-01

**Authors:** Leila Solouki, Soodeh Shahsavari, Hamid Sharini, Amir Hossein Hashemian, Volker Johann Schmid, Samira Jafari, Saleh Rouhi, Behzad Mahaki

**Affiliations:** 1 Student Research Committee, Kermanshah University of Medical Sciences, Kermanshah, Iran; 2 Health Data Science Research Center, Health Research Institute, Kermanshah University of Medical Sciences, Kermanshah, Iran; 3 Department of Biostatistics, School of Health, Kermanshah University of Medical Sciences, Kermanshah, Iran; 4 Department of Biomedical Engineering, Faculty of Medicine, Kermanshah University of Medical Sciences, Kermanshah, Iran; 5 Bioimaging Group, Department of Statistics, Ludwig Maximimilians, Munich, Germany; 6 Modeling in Health Research Center, Institute for Futures Studies in Health, Kerman University of Medical Sciences, Kerman, Iran; 7 Department of Medical Physics, Faculty of Medicine, Kermanshah University of Medical Sciences, Kermanshah, Iran; Al-Nahrain University, IRAQ

## Abstract

**Background:**

Fibromyalgia (FM) is a multifaceted chronic pain disorder presenting with pain throughout the musculoskeletal system, alongside chronic fatigue,sleep disturbances, and cognitive impairments. Current diagnostic criteria primarily rely on symptom questionnaires, with no specific laboratory markers or definitive tests, making diagnosis subjective and often delayed. Moreover, the overlap with other central pain syndromes further complicates accurate diagnosis. Neuroimaging studies, particularly functional magnetic resonance imaging (fMRI), have shown promise in elucidating brain abnormalities underlying FM. However, translating these findings into reliable diagnostic tools remains challenging, especially given small sample sizes. Therefore, this study aims to enhance the diagnostic accuracy of FM based on fMRI data by developing an intelligent model. This model employs machine learning algorithms designed to perform effectively with limited dataset sizes and incorporates features extracted through graph theory methods. We analyzed fMRI data from 32 FM patients and 30 healthy controls. Graph theory metrics were extracted from 13 regions of the emotion regulation network. A genetic algorithm was employed to select the most effective features, which were then used to train multiple machine learning classifiers—including Support Vector Machine (SVM) with polynomial and Radial Basis Function (RBF) kernels, Random Forest, Gradient Boosting Machine, Adaptive Boosting, and SVM with Mixture Kernels—within a cross-validation framework to ensure robustness.

**Result:**

The genetic algorithm selected 84 features as inputs for machine learning algorithms. The polynomial SVM achieved the highest overall accuracy, whereas the SVM with mixture kernels demonstrated superior sensitivity and AUC. Sensitivity and AUC were prioritized as the main criteria for model selection due to their clinical relevance in reducing false negatives.

**Conclusion:**

The study identified the SVM with Mixture Kernels as the most effective model for distinguishing FM patients from healthy controls. This approach demonstrates promising potential for enhancing diagnostic accuracy and supporting psychological and psychiatric interventions.

## Introduction

Fibromyalgia (FM) is a multifaceted chronic pain disorder presenting with extensive pain throughout the musculoskeletal system, alongside chronic fatigue, disruptions in sleep patterns, and cognitive impairments [1]. This disorder affects between 0.2% and 6.6% of the global population, while its prevalence among women has been reported to range from 2.4% to 6.8%. [2]. The multifaceted clinical manifestations of FM present substantial diagnostic and management challenges for both patients and healthcare providers. Despite the widespread occurrence of this disorder, its exact cause remains unknown, as it is shaped by a complex interaction of environmental, biological, and genetic influences [3].

Central sensitization is widely acknowledged as a fundamental mechanism in FM, leading to altered pain perception. Additionally, emerging research suggests that small-fiber neuropathy and stress-related dysautonomia may also contribute to the condition’s development [3]. The lack of specific laboratory biomarkers for diagnosing FM presents significant clinical challenges, rendering diagnosis primarily dependent on comprehensive clinical evaluation, including assessment of pain indices and symptom severity, which complicates accurate and timely identification of the condition [4]. However, the complex nature of FM—characterized by heterogeneous manifestations, unclear boundaries and its overlap with other central pain disorders further complicates diagnosis. This complexity can sometimes lead to misdiagnosis [5].

Since there is no definitive method for diagnosing fibromyalgia, researchers and physicians are seeking more standardized and objective diagnostic evidence to make the diagnosis more scientific. In recent years, advancements in neuroimaging techniques have facilitated investigations into the neural mechanisms associated with FM. These studies have revealed alterations in brain functional connectivity that may be linked to the disorder’s symptoms [6].

Among various neuroimaging techniques, graph theory provides a powerful analytical framework for investigating intricate brain networks, offering critical insights into both network structure and functional connectivity. This approach has been utilized with multiple neuroimaging modalities, including functional MRI (fMRI), electroencephalography (EEG), and magnetoencephalography (MEG), to investigate both stable and dynamic connectivity patterns in individuals diagnosed with FM [7,8]. Among neuroimaging techniques, fMRI has attracted considerable interest from researchers and physicians due to its superior spatial resolution, safety, and ability to evaluate central brain regions effectively [9].

fMRI is capable of detecting brain abnormalities that may not be identified using other imaging modalities, particularly when alterations are subtle and structural changes are minimal or absent. [10]. However, since the protocols for recording and analyzing fMRI data for diagnosing this condition are not yet well defined, combining machine learning techniques with neuroimaging methods may enhance diagnostic accuracy [11].

Machine learning models have the potential to support the development of more efficient diagnostic methods by utilizing information beyond the practical experience of individual physicians [12]. These models typically require large datasets to achieve optimal performance. However, collecting large training datasets is often impractical in specific applications, such as clinical trials and brain imaging, where data collection or processing is prohibitively expensive or logistically challenging. Consequently, small datasets represent a common obstacle for machine learning approaches.

To address this limitation, the application of support vector machines, and boosting models has shown promise in enhancing model generalization and accuracy with limited data [13].

Accordingly, this study aims to employ various machine learning methods (SVM, SVM-MK, RF, GBM, and AdaBoost) based on advanced functional magnetic resonance imaging (fMRI) analysis using graph theory within the context of an emotion regulation task, to improve the diagnostic accuracy of fibromyalgia.

## Materials and methods

### Ethics statement

The present study employed the UCLA Consortium for Neuropsychiatric Phenomics (CNP) dataset, which is publicly available from the OpenNeuro repository (https://openneuro.org/datasets/ds004144/versions/1.0.2/file-display/README). The research was conducted in accordance with the ethical requirements and protocols approved by the Institutional Review Board (IRB) of the University of California, Los Angeles (UCLA).Written informed consent was obtained from all participants prior to their enrollment in the study. To comply with institutional requirements, this study also received ethical approval from the Research Ethics Committee of the National Institute of Psychiatry and from the Ethics Committee of Kermanshah University of Medical Sciences (Approval number: IR.KUMS.REC.1404.048).

### Participants

In this study, fMRI brain images from 62 women, including 32 individuals diagnosed with FM and 30 healthy control subjects, alongside clinical information from participants aged 30–50 years (mean age: 41.73), were analyzed. The data were collected between 23/11/2018 and 18/10/2019. Moreover, the data were accessed for our research purposes on 27 /12/2024. The authors did not have access to any personally identifiable information that could be used to identify participants after data collection. Individuals diagnosed with FM had undergone prior clinical assessment by either an internist or a rheumatologist. To verify the diagnosis, the Widespread Pain Index (WPI) and the Symptom Severity Scale (SSS), introduced by the American College of Rheumatology in 2010, were administered during a session conducted at least two weeks before the scanning session. Furthermore, a physical examination was performed to evaluate tender points, following the diagnostic criteria established by the American College of Rheumatology in 1990. All participants were right-handed and had attained at least an elementary education. Exclusion criteria included a diagnosis of major psychiatric disorders (e.g., obsessive-compulsive disorder, psychosis, or bipolar disorder), neurological conditions, cardiovascular disease, or any other pain disorder presenting with greater pain intensity than FM. Before the MRI scan, participants underwent training for the emotion regulation task and were instructed to refrain from using benzodiazepines or painkillers for at least 24 hours before the session. To better understand, all research steps are shown graphically in Fig 1.

**Fig 1:**
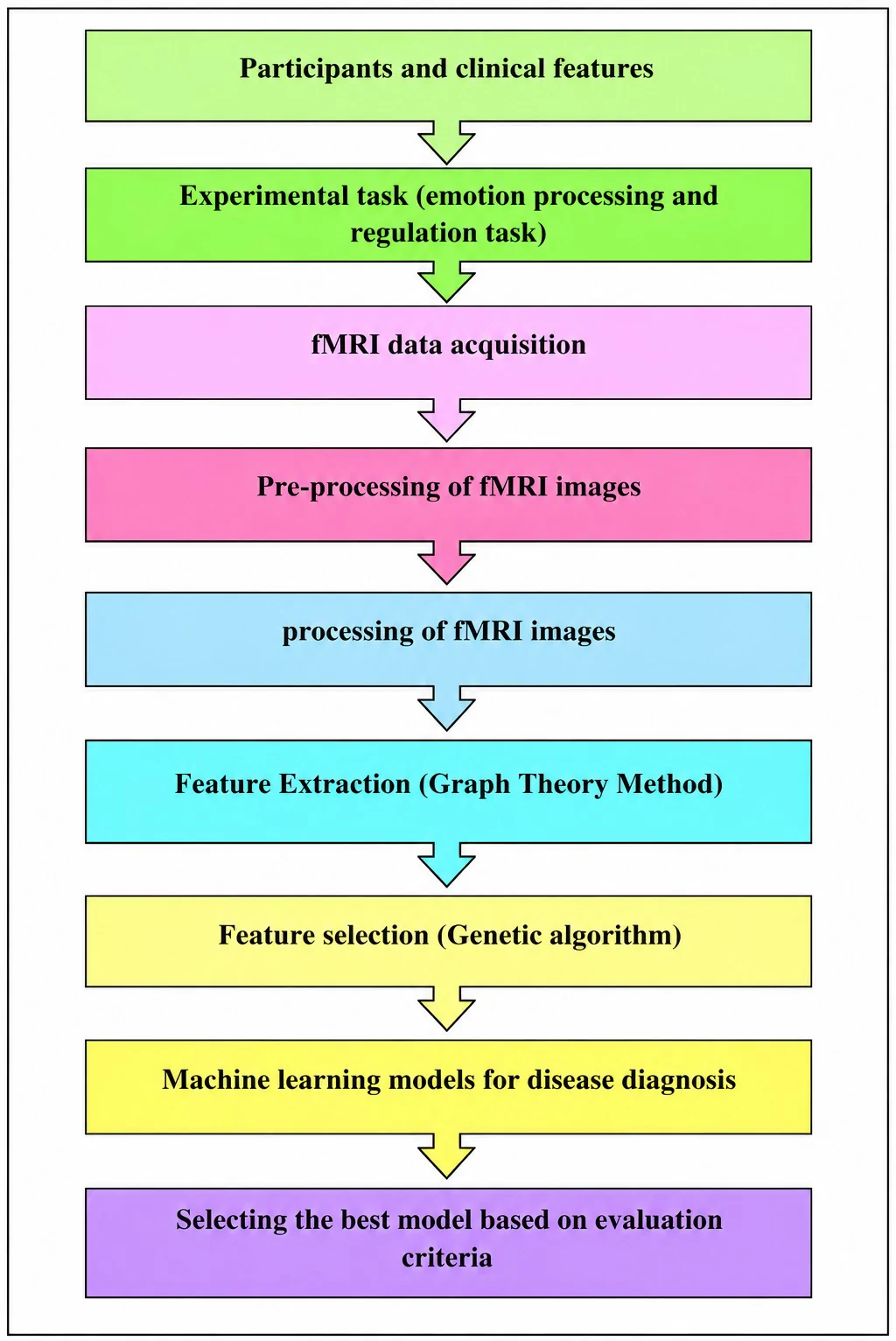
Graphic abstract (steps performed in this study).

### fMRI Measurements

Whole-brain anatomical and functional imaging was performed using a 3.0 Tesla Philips Ingenia MRI system equipped with a 32-channel phased-array head coil. A task-based functional MRI sequence employing Gradient Echo-Planar Imaging was used, with parameters optimized to ensure high-quality data acquisition.

Functional imaging parameters included a repetition time (TR) of 2000 ms and an echo time (TE) of 30.001 ms. To ensure signal stabilization, five dummy scans were acquired prior to data collection. The imaging matrix was set to 80 × 80, with a flip angle (FA) of 75°, and a field of view (FOV) of 240 mm². Voxel dimensions were defined as 3 × 3 × 3 mm. Slices were acquired in an interleaved ascending order, with each slice having a thickness of 3.0 mm. A total of 36 slices were collected per volume. Phase encoding was oriented in the anterior-to-posterior (AP) direction. In total, 834 volumes were acquired over approximately 27.8 minutes.

### Task-based fMRI

In this study, participants engaged in an emotion processing and regulation task. The task involved responding to three categories of stimuli, each selected according to the level of emotional arousal they were intended to evoke: negative (e.g., a distressing image of an accident scene), positive (e.g., an individual celebrating a special occasion with a cake), and neutral (e.g., a picture of a person holding a cup of tea).

Consistent with the study protocol, participants were instructed to carefully observe the presented images and allow themselves to experience any emotional reactions elicited by the stimuli, without trying to control or change their affective responses. The task was repeated across three separate blocks, each consisting of four visual stimuli, resulting in a total of twelve images presented to each participant. The stimuli were selected from the International Affective Picture System (IAPS), most of which depicted scenes involving human subjects for each stimulus type—positive, negative, and neutral [13]. As illustrated in Fig 2, sample images representing neutral, positive, and negative emotional valence are shown for illustrative purposes.

**Fig 2:**
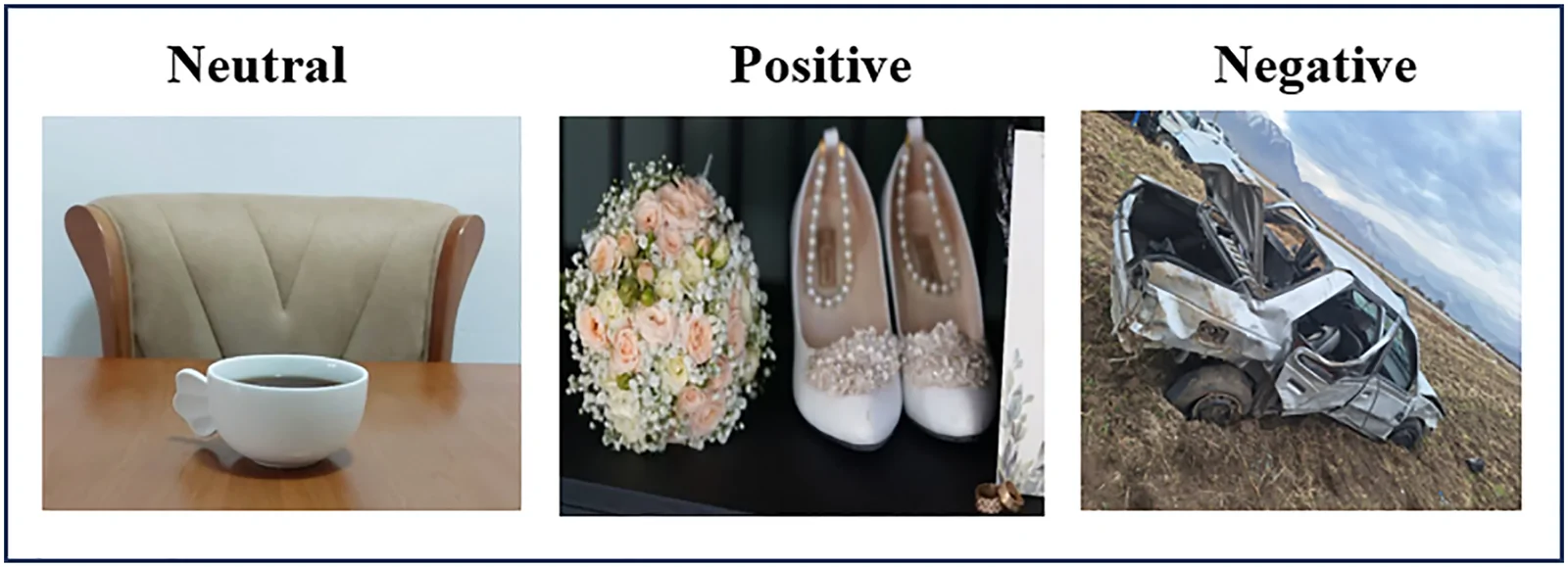
Sample images captured by the authors, similar but not identical to those used in the study, representing neutral (left column), positive (middle column), and negative (right column) emotional valence. These images are provided solely for illustrative purposes.

### Pre-processing procedure

To minimize the influence of scanner-related instability on magnetic moment measurements, the first five frames of each participant’s imaging dataset were excluded from analyses. All preprocessing steps were performed using algorithms implemented in the CONN toolbox within MATLAB (2022b). These steps included field map correction to reduce image distortion, slice timing correction to account for differences in acquisition time across slices, and realigning the entire fMRI dataset to the average volumes to correct for head motion.

Structural and functional images were co-registered to enhance the accuracy of activity localization. Structural images were also segmented to produce bias-corrected anatomical images. Both structural and functional data were then spatially normalized to the standard anatomical space defined by the Montreal Neurological Institute (MNI). During the smoothing stage, a Gaussian filter with a full width at half maximum (FWHM) of 6 mm was applied to the functional images to reduce high-frequency noise [14].

### Regions of Interest (ROI)

A recent meta-analysis by Kohn and colleagues (2014) explored the neural mechanisms implicated in emotion regulation. The findings demonstrated that, during emotion regulation, the angular gyrus (AG), supplementary motor area (SMA), and superior temporal gyrus (STG) are essential for processing information from the frontal cortex. The anterior middle cingulate cortex (aMCC) serves an integrative role in transmitting emotional information and is essential for the generation of affect. The dorsolateral prefrontal cortex (DLPFC) regulates cognitive processes such as attention, while the ventrolateral prefrontal cortex (VLPFC) is involved in signaling salience.

Briefly, the neural pathway proposed suggests that the AG, SMA, STG, and VLPFC receive emotional and arousal information from the amygdala. Appraisal begins in the VLPFC, which assesses the need for emotion regulation. This information is subsequently transmitted to the DLPFC, where the regulation process occurs. Finally, the DLPFC sends signals via the aMCC back to the amygdala, STG, SMA, and AG, leading to physiological and behavioral reactions. A comprehensive reference ring view of all ROIs is presented in Fig 3, along with their corresponding MNI coordinates listed in Table 1 [15]. These regions are displayed in coronal, axial, and sagittal views in Fig 4.

**Table 1:** MNI coordinates of the 13 ROIs relevant to emotion regulation network.

ROI	Abbreviation	MNI coordinate
**Left angular gyrus**	AG l	(−50, −56, 30)
**Right angular gyrus**	AG r	(52, −52, 32)
**Left supplementary motor area**	SMA l	(−5, −3, 56)
**Right supplementary motor area**	SMA r	(6, −3, 58)
**Left superior temporal gyrus**	STG l	(−52, 8, −18)
**Right superior temporal gyrus**	STG r	(52, 8, −18)
**Anterior middle cingulate cortex**	aMCC	(2, 14, 48)
**Left dorsolateral prefrontal cortex**	DLPFC l	(−29, 41, 27)
**Right dorsolateral prefrontal cortex**	DLPFC r	(44, 23, −3)
**Left ventrolateral prefrontal cortex**	VLPFC l	(−44, 23, −3)
**Right ventrolateral prefrontal cortex**	VLPFC r	(25, 2, 0)
**Left amygdala**	amygdala l	(−23, −5, −18)
**Right amygdala**	amygdala r	(23, −4, −18)

**Fig 3:**
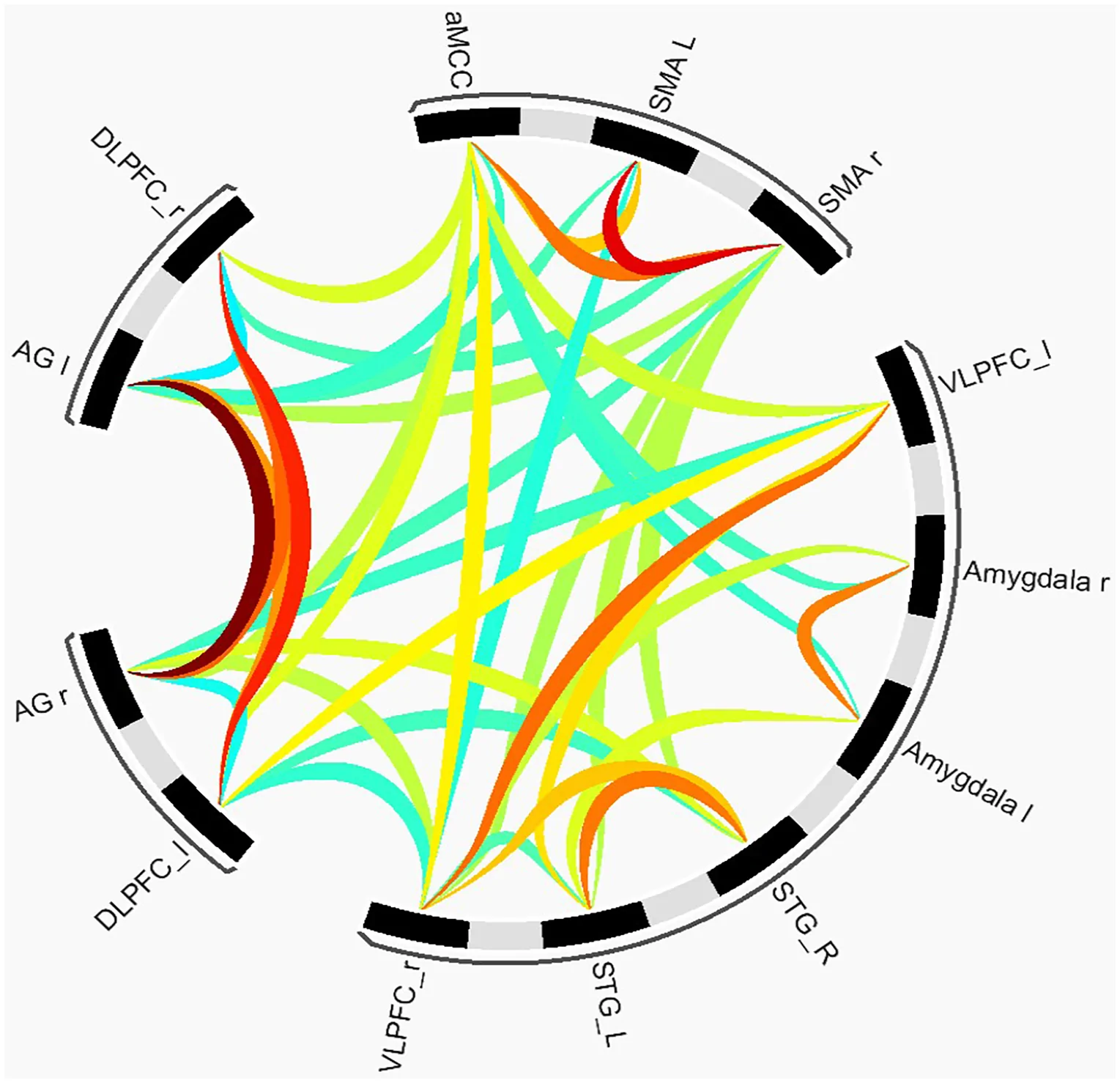
A reference ring view illustrating the full set of ROIs constituting the emotion regulation network.

**Fig 4:**
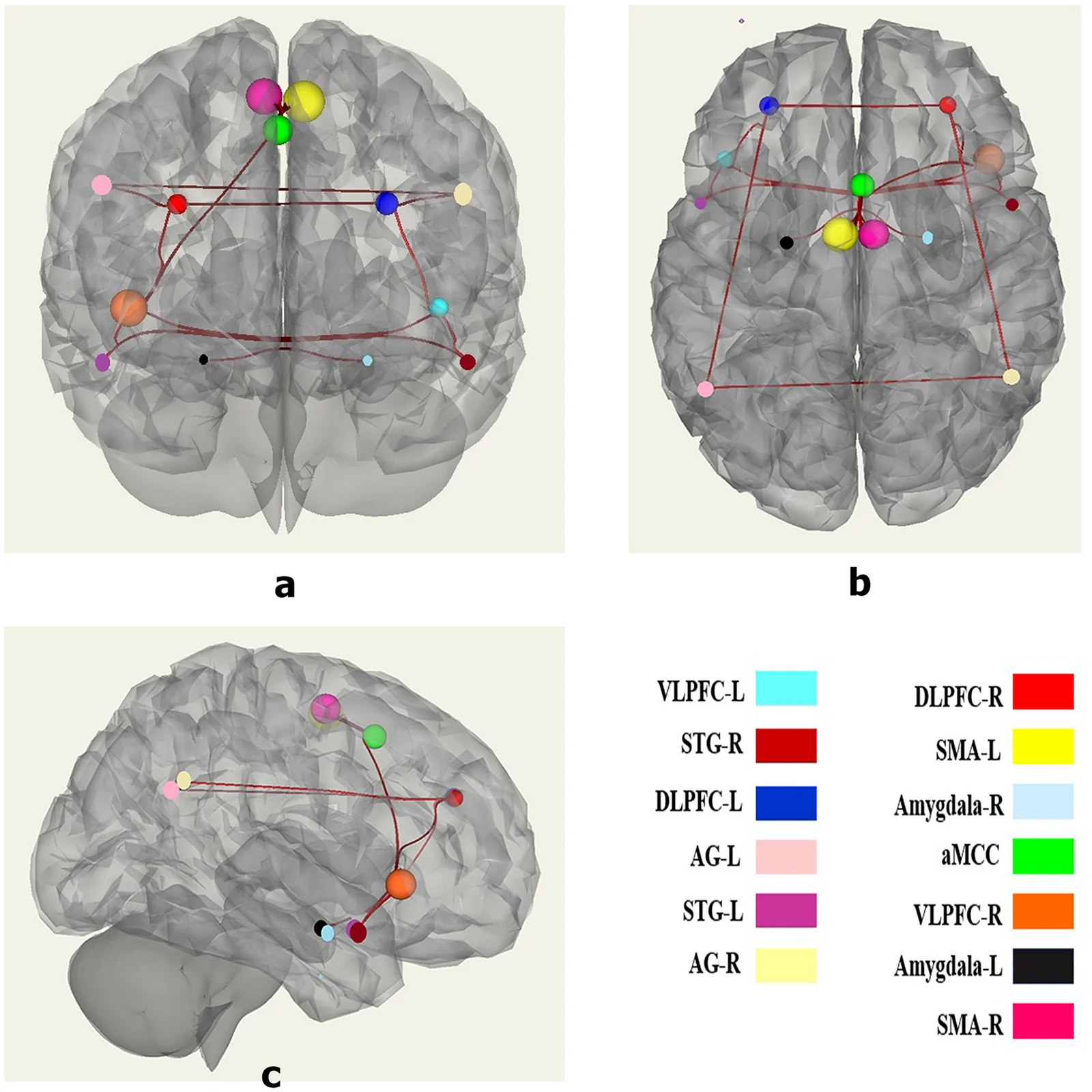
(a, b, and c) 3D views of coronal, axial, and sagittal planes of the brain’s emotion regulation network regions.

### Investigated variables

After pre-processing using the graph theory method, the graph metrics of Global Efficiency (reflecting the closeness of a node to all others), Average Path Length (The mean shortest path length from a given node to all other nodes in the network), Betweenness Centrality (Identifies the most influential network nodes), Local Efficiency (describes how neighbors in a specific region of the network are functionally connected), Degree (The total number of direct connections (edges) a node has with other nodes in the network), and Clustering Coefficient (The proportion of connected nodes among all neighboring nodes) were computed for each node (13 targeted brain regions) based on the adjacency matrix. These metrics were calculated across three task conditions: positive, neutral, and negative. Additionally, clinical variables including age, marital status, education level, occupation, and years of education were evaluated [16].

### Graph theory method

In recent years, graph-theoretical approaches have gained prominence in neuroscience, offering robust tools to characterize neural relationships and delineate structural and functional alterations across healthy and diseased groups. Graph-theoretical metrics enable systematic analysis of brain connectivity at both local (regional) and global (network-wide) levels. Graph theory methods can provide important new insights into the structure and function of networked brain systems, including their architecture, evolution, development, and clinical disorders [17].

Graphs are a collection of elements (nodes or vertices) and their binary connections, known as edges. In their most basic representation, these relationships can be captured using a connection (adjacency) matrix. The full set of pairwise connections defines the graph’s topology, illustrating the complete pattern of connectivity among nodes and edges. In whole-brain networks derived from neuroimaging data, nodes typically correspond to brain regions, while edges represent, structural, functional or affective connectivity [18].

Thirteen brain regions implicated in emotion regulation among FM patients were selected for analysis in this study [15]. Subsequently, graph theory analysis was performed using the CONN toolbox in MATLAB (2022b) to compute the features related to the distribution of neighborhoods and connections in these brain regions.

### Feature selection using genetic algorithm

Feature selection is crucial in machine learning due to the large number of features, many of which may not carry much information. The elimination of redundant or irrelevant features is important to obtain a subset with minimal relevant features, thereby building models with reduced computational complexity, and improving classification accuracy. In this study, the genetic algorithm (GA) was employed for feature selection due to its ability to efficiently explore large, complex, and high-dimensional feature spaces, which is critical when handling datasets with many variables but relatively small sample sizes, such as our dataset comprising 278 features and 62 participants. Unlike traditional linear methods, GA uses a population-based, evolutionary strategy that can capture complex non-linear interactions and dependencies among features, improving the selection of informative features while excluding irrelevant or redundant ones [19]. Furthermore, GA’s robustness against getting trapped in local optima enables better exploration of the search space, making it particularly useful for medical imaging applications and biomedical data [20]. As a result, GA provides a powerful and flexible heuristic approach that enhances model performance by optimizing the feature subset in challenging high-dimensional settings. In the present study, genetic algorithm–based feature selection was performed within each fold of the repeated 10‑fold cross‑validation with 100 repetitions. In each fold, feature selection was restricted to the training set (9 folds), and the selected features were subsequently applied to the corresponding test set (1 fold). This nested procedure ensured that no information from the test data was used during feature selection, thereby preventing data leakage and avoiding artificially optimistic performance estimates.

### Statistical analysis

Descriptive statistics were applied to analyze the data, using frequency tables for qualitative variables and measures of central tendency and dispersion indices for quantitative variables. This approach sought to describe the most significant characteristics of the gathered data.

### Cross-validation

In the present study, 10-fold cross-validation was employed to assess the performance of the classification algorithms, thereby preventing overfitting. Specifically, the subject dataset was randomly segmented into ten parts, with nine parts considered as the training set and the remaining part regarded as the testing set. To enhance result reliability, 10-fold cross-validation was repeated 100 times in this study. The final result was obtained by averaging the outcomes across 100 repetitions.

### Machine learning algorithms

This study initially used five machine learning models: SVM (RBF), SVM (Poly), RF, GBM, and AdaBoost. Subsequently, an SVM model with a mixed kernel was employed. Finally, these models were compared using appropriate criteria to determine the best model.

### Support Vector Machine (SVM)

The SVM technique is a highly effective machine learning approach and one of the supervised learning methods applied to classification and regression tasks. It is known as a small sample learning method with a strong theoretical base because its temporal and spatial complexities make it unsuitable for large datasets [21]. As a result, SVM has become very popular for analyzing low-sample-size data, including psychiatric and neuroimaging data, particularly for applications involving diagnosing and predicting brain diseases, where it performs well.

### Adaptive Boosting (AdaBoost)

AdaBoost, short for Adaptive Boosting, is a popular ensemble learning algorithm that sequentially combines weak classifiers to construct a highly accurate predictive model, focusing on misclassified samples in subsequent iterations. The final prediction is derived by combining the outputs of all weak classifiers, where each classifier’s influence is weighted according to its performance. The AdaBoost method offers several key benefits, including high accuracy, ease of implementation, and a decrease in overfitting [22].

### Random Forest algorithm

The Random Forest algorithm is a robust supervised learning method that utilizes an ensemble of decision trees to enhance predictive accuracy and control overfitting. Random Forest comprises multiple decision trees, where each decision tree selects its discriminative features from a bootstrap training set Si, with *i* representing the *i*th internal node. In Random Forest, decision trees are constructed using the Classification and Regression Tree (CART) algorithm without pruning. As the number of trees in the forest becomes extremely large, the generalization error also increases until it converges to a certain boundary level. In classification tasks, the final prediction is obtained through majority voting across the ensemble of trees. This approach allows Random Forest to effectively manage complex datasets, making it widely applicable in finance, healthcare, and marketing domains, where high dimensionality and noise are common challenges. This method has shown excellent performance in multiple applications, particularly when dealing with high-dimensional data and small sample sizes [23].

### Gradient Boosting Machines (GBMs)

Gradient Boosting Machines (GBMs) are robust machine learning techniques with wide-ranging applications. They are an ensemble technique that merges several weak prediction models, typically decision trees, to form a strong predictive model. The essential concept of GBM involves iteratively building an ensemble of weak models, where each new model seeks to correct the mistakes of its predecessors. The models are built sequentially, with each new model paying more attention to the data points that the previous models incorrectly predicted. GBM models have become powerful tools for predictive tasks due to their flexibility, high predictive power, and ability to manage complex interactions between variables, particularly in both balanced datasets and small datasets [24].

### Support vector machine with mixture kernels (SVM-MK)

A challenging problem in SVM is the selection of the kernel function, which measures the similarity between two vectors. Various kernel functions have been suggested for SVM, with the most common being the RBF and polynomial kernels, which have been widely used for class separation [25]. The RBF kernel is a local kernel function with strong learning capacity but limited generalization performance, whereas the polynomial kernel is a global kernel function with opposite characteristics. To address the limitations of each kernel function, the SVM method with a mixed kernel function, combining polynomial and RBF kernels, can be used.

### Compare the performance of models

In the present study, after fitting the algorithms, we utilized the metrics of accuracy, specificity, sensitivity, the area under the ROC curve, and F1 score to evaluate their performance. The chosen machine-learning model was fitted for training set in each 10-fold, and performances were evaluated on the test set. Accuracy is one of the criteria used to assess the overall effectiveness of a model, which, in the present study, reflects the model’s ability to distinguish FM from HC correctly. However, in the context of a clinically applicable diagnostic screening tool, greater emphasis was placed on sensitivity and AUC. Sensitivity represents the proportion of true positive cases (FM) correctly identified by the classifier and is particularly important for minimizing false negatives in clinical screening, whereas specificity denotes the percentage of correct negative cases (HC) correctly diagnosed by the classifier. Finally, the area under the performance characteristic curve (AUC) was used as a criterion to measure the discrimination ability of a diagnostic test or prediction model. Therefore, in this study, AUC and sensitivity were considered the primary criteria for model evaluation and selection, whereas accuracy, specificity, and F1 score were reported as complementary performance measures. Models with an AUC of less than 60% do not perform well, while models with an AUC greater than 80% perform very well and are better for predicting different classes [26]. A paired t‑test was used to statistically compare the performance of the two best‑performing models. Model evaluation was based on 10‑fold cross‑validation repeated 100 times. For each repetition, the performance metrics (AUC and sensitivity) were averaged across the 10 folds, yielding 100 paired performance estimates. The paired t‑test was then applied to determine whether the differences between the two top models were statistically significant. All analyses were conducted using MATLAB (version 2022b) and Python, with a significance level 0.05.

## Results

The information from 62 women, including 32 individuals diagnosed with FM and 30 healthy individuals, alongside clinical information for individuals aged between 30 and 50 years (mean age: 41.73), has been studied (Table 2). The mean age of participants in both groups is very similar, with a comparable standard deviation, indicating age-matched groups. Most participants in both groups are married (HC: 63.6%, FM: 51.5%). The FM group has a slightly higher percentage of singles and widows compared to HC and Divorced/separated percentages are similar. Marital status distribution is broadly similar, with minor differences. The FM group tends to have slightly lower employment rates and education levels. These demographic characteristics provide context for comparing other study outcomes between healthy controls and fibromyalgia patients.

**Table 2:** Demographic characteristics of participants in each group.

Variable	FM	HC
**Age, mean (SD)**	41.78 (6.18)	40.97 (6.0)
**Marital status, n**		
**Single**	9 (28.1%)	7 (23.33%)
**Married/cohabitating**	17 (53.1%)	19 (63.33%)
**Divorced/separated**	4 (12.5%)	3 (10%)
**Widow**	2 (6.3%)	1 (3.34%)
**Education, n (%)**		
**Elementary**	2 (6.3%)	1 (3.3)
**High school or technical**	10 (31.3%)	7 (23.3)
**Bachelor**	13 (40.6%)	14 (46.7)
**Postgraduate**	**7 (**21.8%)	8 (26.7)
**Years of study, mean (SD)**	15.5 (4.02)	16.76 (4.02)
**Occupation, n (%)**		
**Employed**	18 (56.2%)	22 (73.3)
**Unemployed/Housewife**	11 (34.4%)	7 (23.4)
**Student**	3 (9.4%)	1 (3.3)

FM: Fibromyalgia, HC: healthy controls

### Results of feature selection

In the present study, due to the large number of features (278) and also some demographic variables, a genetic algorithm was used to reduce computational complexity, improve classification accuracy, and select the most effective features. As expected, demographic variables were discarded as the main features, which were shown in the previous section to be relatively similar in the two groups. The number of 50 subsets (chromosomes) was considered the initial population, and the fitness index was used to determine the value of each chromosome, which is the accuracy value obtained from the classification of the random forest algorithm. The selection of features was calculated using the genetic algorithm in the third iteration, achieving 85.8% accuracy. Finally, 84 practical and vital features, were selected as input features for machine learning algorithms. For greater transparency and reproducibility, the complete list of these features is presented in Table 3, which specifies the graph metric, the corresponding brain region (ROI), and the stimulus category (Happy, Neutral, or Negative) associated with each feature.

**Table 3:** Selected features (graph metrics, brain regions, and stimulus conditions) identified using the Genetic Algorithm.

Metrics / Stimulus Category	Selected regions		
	Happy	Negative	Neutral
**Degree**	VLPFC l, VLPFC r, STG l, aMCC	AG l, Amygdala l	DLPFC l, VLPFC r
**Betweenness Centrality**	SMA r, AG l, STG l, STG r	STG l, DLPFC r, VLPFC l, STG r	
**Cost**	VLPFC r, DLPFC l, VLPFC l, aMCC, Amygdala r, SMA r, STG r, Amygdala l, SMA l, AG r	VLPFC r, STG l, DLPFC l, aMCC, SMA r, SMA l, AG r	STG r, DLPFC r, aMCC
**Global Efficiency**	STG l, aMCC, Amygdala r, SMA r	SMA r, SMA l, VLPFC r, VLPFC l	VLPFC r, VLPFC l, Amygdala r
**Local Efficiency**	STG l, DLPFC l, SMA r, SMA r, VLPFC r, STG r	aMCC, AG l, AG r, DLPFC l	VLPFC l, STG l, aMCC
**Clustering Coefficient**	DLPFC l, DLPFC r, SMA l, aMCC, STG r	DLPFC l, VLPFC r, VLPFC r, SMA l, AG r	VLPFC r, DLPFC r, DLPFC l, Amygdala r
**Average Path Length**	VLPFC r, VLPFC l, DLPFC l, AG r, Amygdala l	STG r, DLPFC l, AG r	DLPFC l, Amygdala l

r: Right, l: Left

As expected, demographic variables including age, marital status, education level, occupation, and years of schooling were also incorporated as input features for the machine learning models.

### Performance of the methods

The performance of the models was evaluated using sensitivity, specificity, AUC, F1‑score, and accuracy. As defined a priori in the Methods section, model selection prioritized maximizing AUC and sensitivity, as these metrics are more important than raw accuracy for a clinically applicable diagnostic screening tool due to their role in minimizing false‑negative results. Although the SVM (Poly) model achieved the highest accuracy, it was not considered the optimal model based on the predefined priority criteria. According to these criteria, the SVM‑MK model demonstrated better overall diagnostic performance and was therefore selected as the optimal model for distinguishing FM from HC.

This model demonstrated the highest accuracy (80%), AUC (80%), and specificity (70%), reflecting its superior overall performance. Furthermore, its high sensitivity (90%) indicates a strong ability to accurately identify individuals with FM. The highest F1-score (82%) also indicates a well-balanced trade-off between precision and recall.

Following the SVM-MK model, the SVM with the polynomial kernel model performed best (ACC = 84%, SE = 72%, SP = 75%, F1 score = 76%, and AUC = 0.74%). This model demonstrates high accuracy, with moderate specificity and sensitivity, and its F1 score indicates satisfactory performance. Also, the SVM (RBF), AdaBoost, RF, and GBM models ranked in order of performance, respectively. The SVM model with RBF kernel achieved an acceptable accuracy of 0.76 and an AUC of 0.66. While the sensitivity was relatively moderate at 0.73, the specificity was lower at 0.60, indicating that the model performs well in identifying FM patients but is less effective in correctly classifying HC. The F1 score of 0.69 reflects a reasonable balance between precision and recall. The AdaBoost model demonstrated moderate performance, with an accuracy and AUC of 0.68. It achieved a sensitivity of 0.56, indicating a moderate ability to identify FM patients, and a relatively high specificity of 0.80, reflecting stronger performance in recognizing HC cases. The F1 score of 0.66 suggests a reasonably balanced classification outcome. In the RF model, both accuracy and AUC were 0.63. The sensitivity was moderate at 0.56, while the specificity was relatively higher at 0.70, indicating better performance in identifying HC. The F1 score of 0.62 reflects a moderate level of overall classification performance. In the GBM, Lowest accuracy (0.54) and AUC (0.56). Sensitivity (0.60) is relatively good but specificity is poor (0.50), indicating many false positives. F1 score (0.55) is the lowest among the better-balanced models (Table 4). Fig 5 shows the ROC diagram for each of the models.

**Table 4:** The results of comparing mean of the performance indices in test set of 10-fold cross validation of ML Models.

Models	Accuracy (mean ± SD)	AUC (mean ± SD)	Sensitivity (mean ± SD)	Specificity (mean ± SD)	f1_ Score (mean ± SD)
**AdaBoost**	0.68 ± 0.04	0.68 ± 0.03	0.56 ± 0.05	0.80 ± 0.03	0.66 ± 0.04
**SVM (RBF)**	0.76 ± 0.03	0.66 ± 0.04	0.73 ± 0.03	0.6 ± 0.04	0.69 ± 0.03
**SVM (Poly)**	0.84 ± 0.02	0.74 ± 0.03	0.72 ± 0.03	0.75 ± 0.03	0.76 ± 0.02
**SVM -MK**	0.80 ± 0.02	0.80 ± 0.02	0.90 ± 0.02	0.70 ± 0.03	0.82 ± 0.02
**Gradient Boosting**	0.54 ± 0.05	0.56 ± 0.04	0.6 ± 0.05	0.5 ± 0.05	0.55 ± 0.04
**Random Forest**	0.63 ± 0.04	0.63 ± 0.03	0.56 ± 0.04	0.70 ± 0.03	0.62 ± 0.04

**Fig 5:**
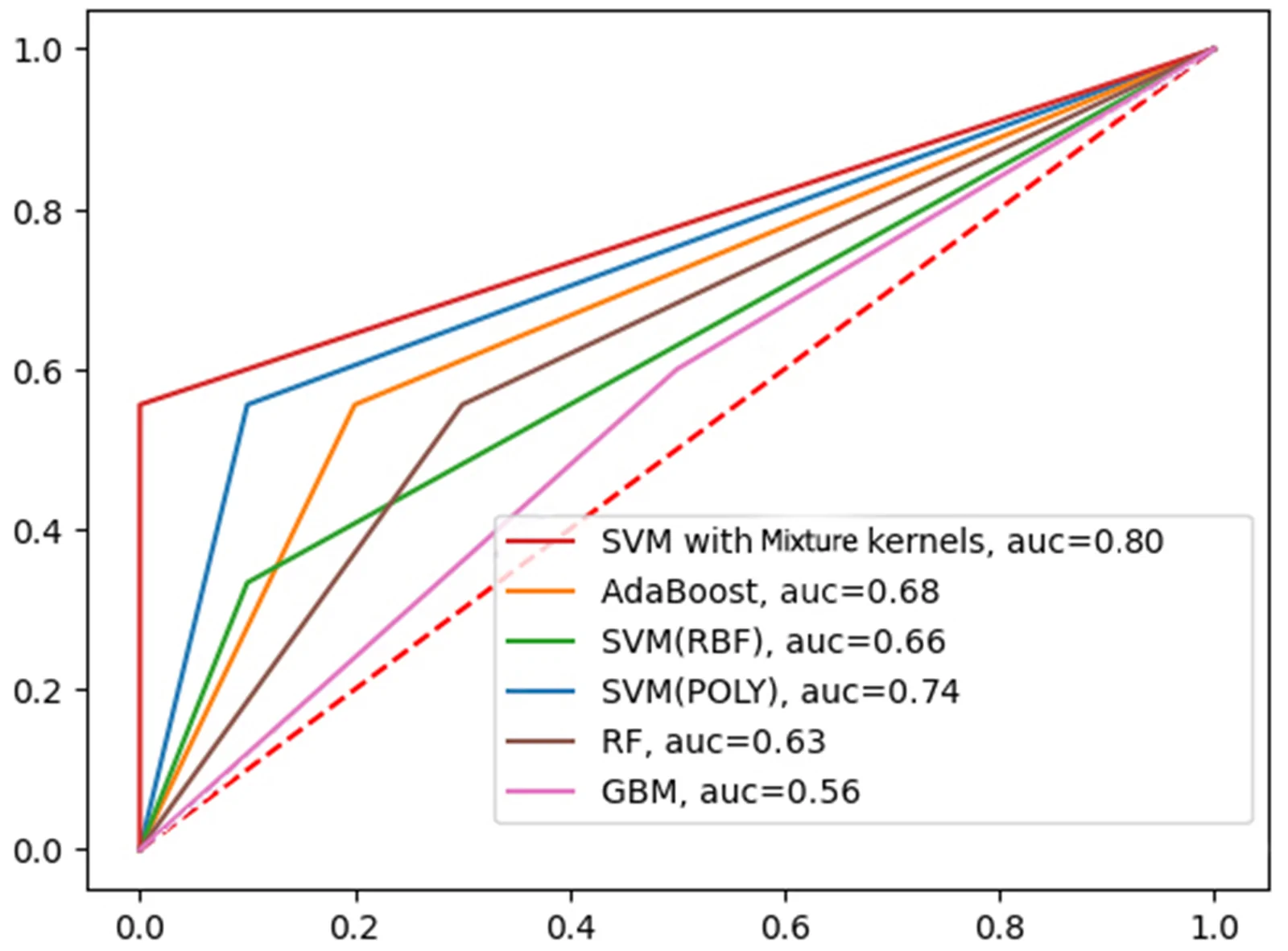
ROC curve for the models used.

To assess the statistical significance of performance differences between the top models, a paired t‑test was conducted across 100 cross‑validation repetitions. This test was applied to the two primary evaluation metrics, AUC and sensitivity. The results indicated that the SVM‑MK model performed significantly better than the SVM‑Poly model. Specifically, the difference in mean AUC between the two models was statistically significant (t = 2.71, p = 0.01), and the difference in mean sensitivity was likewise statistically significant (t = 3.52, p = 0.001) (Table 5).

**Table 5:** Paired t‑test comparison of SVM‑MK and SVM‑Poly classifiers across 100 cross‑validation repetitions.

Metric	SVM‑MK	SVM‑Poly	t-value	p-value
**AUC**	0.80	0.74	2.71	0.01
**Sensitivity**	0.90	0.72	3.52	0.001

## Discussion

This study utilized machine learning techniques and advanced analysis of functional magnetic resonance imaging (fMRI) using graph theory to differentiate between individuals with FM and healthy individuals. After preprocessing and processing the fMRI data using MATLAB, a genetic algorithm was employed to select practical and significant features for disease diagnosis. These selected features were subsequently used as input for various models to enhance diagnostic accuracy. Machine learning algorithms exhibit distinct strengths and limitations in classification tasks, with their accuracy being contingent upon factors such as sample size and dataset complexity. Due to the limited sample size in this study, we utilized machine learning approaches suitable for small datasets. In this study, model selection was guided by the a priori–defined priority of maximizing AUC and sensitivity, given their importance in minimizing false‑negative outcomes in a clinical screening context. Although the SVM (Poly) model achieved the highest accuracy (ACC = 84%, SE = 72%, SP = 75%, F1 score = 76%, AUC = 0.74), it was not considered the optimal classifier under these predefined criteria. Consistent with this rationale, the SVM‑MK model demonstrated superior overall diagnostic performance (AUC = 0.80, SE = 90%, SP = 70%, ACC = 80%, and F1 score = 82%), outperforming SVM (Poly) and the other evaluated models. These findings support the selection of SVM‑MK as the most effective model for distinguishing FM from HC. The SVM(RBF), AdaBoost, RF, and GBM models also performed better.

Aligned with the objectives of our study, the following research focuses on distinguishing FM from HC using ML models based on MRI data. Behr et al. achieved 84.1% accuracy in differentiating FM patients from HC using MRI images and support vector machine (SVM) models [27]. Lopez-Sola et al. demonstrated that machine learning applied to multisensory-stimulated fMRI patterns can effectively differentiate FM patients from pain-free controls [28]. A study by Thanh Nhu et al., using the SVM approach and a combination of network-based resting-state functional connectivity (rs-FC) and brain structural data, achieved 95% accuracy in differentiating FM patients from HC [29].

Studies have also examined machine learning (ML) models for diagnosing other brain diseases using MRI data. In the study by Battineni et al., supervised machine learning techniques, such as RF, Logistic Regression (LR), SVM, and NB, alongside ensemble models such as GBM and AdaBoost—were employed to differentiate AD patients from HC using MRI data. The findings demonstrated that boosting techniques outperformed supervised models; in particular, the Gradient Boosting technique achieved superior classification accuracy (97.58%) compared to other models, while the Naive Bayes (NB) model had the lowest accuracy (95.96%) [30].In contrast to this study’s results, our findings indicate that the SVM-MK and SVM-Poly models performed better than others, including boosting methods.

In our study, the best performance in disease detection was achieved by the SVM-MK model, which combines RBF and polynomial kernels of SVM. The following studies support the superiority of the SVM-MK model. A study by Jeong et al. employed multimodal neuroimaging data from 28 individuals diagnosed with Internet Gaming Disorder (IGD) and 24 healthy controls to evaluate the diagnostic utility of machine learning models. Specifically, SVM, Random Forest, Boosting, and SVM-MK algorithms were utilized for the classification of IGD. The results of this investigation demonstrated that the SVM-MK model exhibited superior classification accuracy (86.5%) in comparison to the other evaluated models [31]. The survey by Tania, Shill, and colleagues reported higher performance of SVM models using combinations of polynomial and RBF kernels, as well as linear and RBF kernels, compared to SVM models with single kernels for breast cancer and cardiovascular disease datasets. Simulation outcomes also supported their results [32]. Tian et al.’s study on the Berg dataset demonstrated that the SVM-MK model achieved superior classification accuracy compared to the single-kernel SVM models using the RBF and polynomial kernels, with accuracies of 50.3%, 48.8%, and 46.8%, respectively [33]. This indicates that integrating multiple kernels can enhance the model’s ability to capture complex data patterns, improving classification performance. Similarly, Song et al.’s research comparing SVM-RBF, SVM-Linear, and SVM-MK models across four datasets showed that SVM-MK outperformed other SVM models, exhibiting better learning ability and higher generalization [34]. In the study by Jerop et al., an SVM model combining three kernels—linear, RBF, and polynomial—was employed to enhance classification accuracy for disease diagnosis across seven medical datasets. The results demonstrated that the combined-kernel SVM model outperformed single-kernel SVM models in terms of accuracy [35]. Most previous neuroimaging work on FM has investigated changes in resting-state connectivity and network properties in FM based on various machine learning models, which often suffer from sample size limitations. The present study adds to the existing literature by focusing on task-based fMRI with graph theory features, which may capture emotion regulation network dysfunctions associated with FM symptoms [36]. The observed superiority of SVM-MK over more conventional kernels and ensemble methods is consistent with prior reports in biomedical ML where hybrid kernels can balance local detail and global structure when data are limited and features are heterogeneous [37]. This study addresses a challenging clinical question where objective, imaging-based biomarkers can be used to complement symptom-based subjective diagnoses of FM. The use of graph theory features derived from emotion regulation networks reflects a plausible pathophysiological hypothesis for FM, which is consistent with previous work demonstrating altered functional connectivity and network properties in FM patients.

This study’s limited number of participants, due to cost and ethical constraints, represents a key limitation. To enhance interpretability, Table 6 provides a comparative summary of our findings alongside previous machine learning studies. Another major limitation is the absence of external validation using an independent cohort. Future investigations with larger and more diverse datasets, as well as independent cohorts, are essential to confirm the robustness, reproducibility, and generalizability of the proposed model.

**Table 6:** Comparison of the present study’s findings with previous machine learning studies.

Study	Sample Size	Imaging Modality	ML Models	The best model
**Present study**	32 FM / 30 HC	fMRI	GBM, RF, AdaBoost, SVM-RBF, SVM-Poly, SVM-MK	SVM-MK (AUC = 80%, SE = 90%, ACC = 80%, F1_ Score = 82%)
**Behr et al.**	17 FM / 17 HC	Structural MRI	SVM, RF, KNN, LR	SVM ACC = 84.1%
**Lopez-Sola et al.**	37 FM / 35 HC	fMRI	MVPA‑based classifiers	SE ≈ 92%, SP ≈ 94%
**Thanh Nhu et al.**	28 FM / 28 HC	Resting-state fMRI +structural MRI	SVM	ACC = 95%, SE = 93%, SP = 96%
**Battineni et al. (AD study)**	200 AD / 28 HC	MRI	RF, LR, SVM, NB, GBM, AdaBoost	GBM (ACC = 97.58%, PRE = 97.60%, F1-score = 97.55%)
**Jeong et al. (IGD study)**	28 IGD / 24 HC	multimodal fusion: PET + EEG + clinical features	SVM (Linear, RBF, Polynomial), RF, Boosting, SVM-MK	SVM-MK (ACC = 86.5%, SE ≈ 85–87%, SP ≈ 86–88%)
**Tania & Shill**	UCI datasets	–	SVM‑Linear, SVM‑RBF, SVM‑Poly, SVM-MK	SVM-MK (ACC ≈ 98%, PRE ≈ 97–98%, SE ≈ 97–98%, F1‑score ≈97–98%)
**Tian et al.**	Standard image datasets (Corel, Caltech, ORL)	–	SVM- RBF, SVM‑Linear, SVM-Poly, SVM-MK	SVM-MK (ACC ≈ 95–97%)
**Song et al.**	UCI datasets (Iris, Wine, Breast Cancer)	–	SVM-RBF, SVM-Linear, SVM-MK	SVM-MK (ACC ≈ 96–98%)
**Jerop et al.**	UCI datasets (Breast Cancer, Heart Disease, Diabetes)	–	SVM- RBF, SVM‑Linear, SVM-Poly, SVM-MK	SVM-MK (ACC = 98.7%, PRE ≈ 98–99%, SE ≈ 98–99%, F1‑score ≈ 98–99%)

FM: Fibromyalgia, HC: Healthy Controls, AD: Alzheimer’s Disease, IGD: Internet Gaming Disorder, SVM: Support Vector Machine, SVM‑MK: Support Vector Machine with Mixed Kernel, RF: Random Forest, GBM: Gradient Boosting Machine, AdaBoost: Adaptive Boosting, KNN: K‑Nearest Neighbors, LR: Logistic Regression, NB: Naïve Bayes, MVPA: Multivoxel Pattern Analysis, ACC: Accuracy, SE: Sensitivity, SP: Specificity (True Negative Rate), AUC: Area Under the ROC Curve, PRE: Precision.

## Conclusion

Among the evaluated models, the SVM-MK demonstrated the best overall performance, achieving the highest AUC, along with high accuracy and sensitivity, moderate specificity, and a strong F1 score. These results highlight its superior ability to accurately distinguish individuals with FM from HC. Other SVM variants, particularly the polynomial kernel SVM, also showed strong performance, highlighting the effectiveness of kernel-based methods in capturing complex patterns in the data. In contrast, models like Gradient Boosting and RF exhibited lower sensitivity or specificity, resulting in reduced overall effectiveness. These findings suggest that leveraging advanced kernel techniques in SVM can significantly enhance classification performance in this context. Future work could focus on further optimizing these models or combining them with other approaches to improve sensitivity without compromising specificity.
